# Supplementary material for: Impact of artificial light at night on diurnal plant-pollinator interactions
Source: Nat Commun. 2021 Mar 16;12:1690. doi: 10.1038/s41467-021-22011-8 (PMC7966740; doi:10.1038/s41467-021-22011-8)

SUPPLEMENTARY INFORMATION for

Impact of artificial light at night on diurnal plant-pollinator interactions

Simone Giavi[1], Colin Fontaine[2], Eva Knop[1,3*]

1 Agroscope, Reckenholzstrasse 191, 8046 Zürich, Switzerland

2 Centre d'Ecologie et des Sciences de la Conservation, CESCO, Muséum National d'Histoire Naturelle – CNRS – Sorbonne Université, 43 Rue Buffon, 75005 Paris, France

3 University of Zürich, Departement of Evolutionary Biology and Environmental Sciences, Winterthurerstrasse 190, 8057 Zürich, Switzerland

*email: eva.knop@ieu.uzh.ch

This PDF file includes:

Supplementary Tables and Figures

Supplementary Table 1: Information on the experimental sites
Supplementary Fig. 1: Raw data per treatment level
Supplementary Fig. 2: Overview light intensity gradient on treatment sites

Supplementary Table 1. Information on the experimental sites. Information on the geographical coordinates, applied treatment (dark control versus experimentally illuminated), the site pair (six pairs), and the number of sampling events.

Site pair	Treatment	North	East	Number of samplings
**1**	Dark	46.66831	7.61394	6
**2**	Dark	46.64352	7.5676	6
**3**	Dark	46.62537	7.56152	6
**4**	Dark	46.60872	7.521	7
**5**	Dark	46.79235	7.40605	6
**6**	Dark	46.77987	7.47612	6
**1**	Illuminated	46.66322	7.61897	6
**2**	Illuminated	46.65197	7.57425	6
**3**	Illuminated	46.60927	7.55803	6
**4**	Illuminated	46.62269	7.54794	7
**5**	Illuminated	46.7977	7.40443	6
**6**	Illuminated	46.78254	7.4601	6

Supplementary Fig. 1. Raw data per treatment level. Mean values +/- SEM of the number of diurnal plant-pollinator interactions over all sampling events per treatment (dark control: gray; illuminated: white) for each selected plant species. Diurnal plant-pollinator interactions (N=2384) were collected in 2016 on 12 ruderal meadows (six exposed to artificial light at night and six left dark as control) sampled six or seven times each. Individual data points are shown in grey. Source data are provided as a Source Data file.

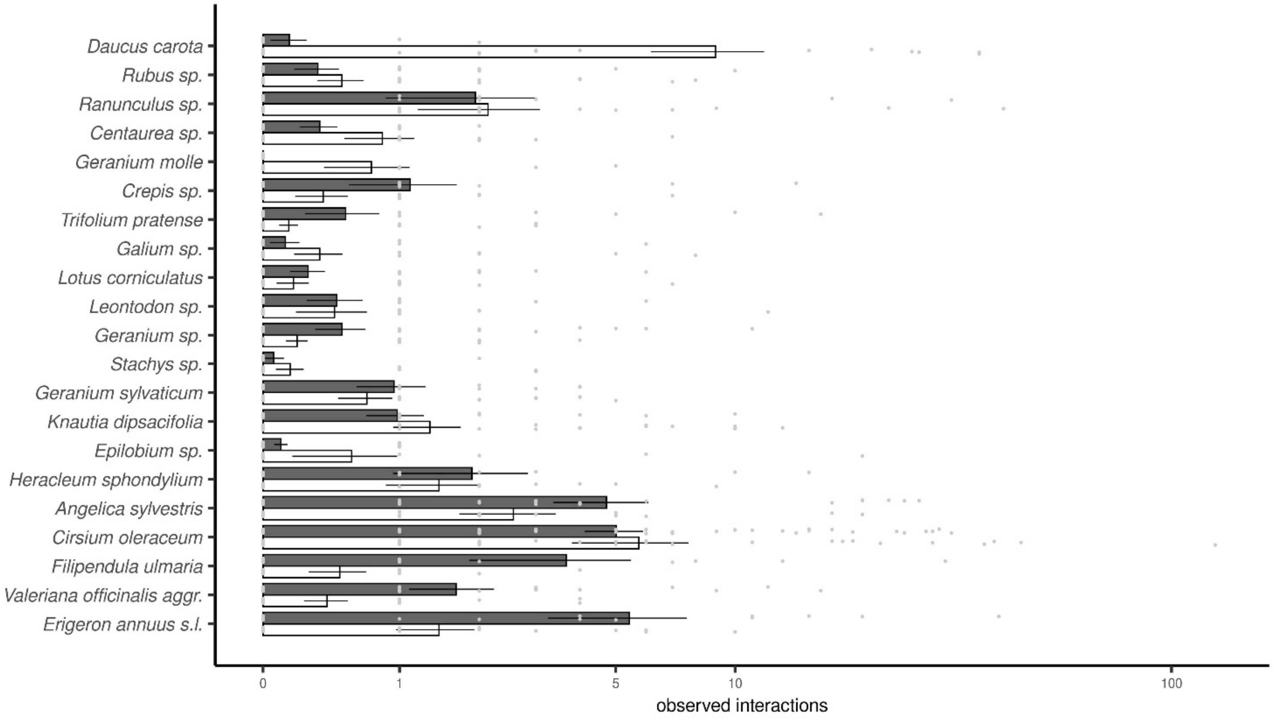

Supplementary Fig. 2. Overview light intensity gradient on treatment sites. Light intensity measurements taken at three illuminated sites (dots, triangles and squares) at each meter from 0 to 60 meters from the lamp. Source data are provided as a Source Data file.

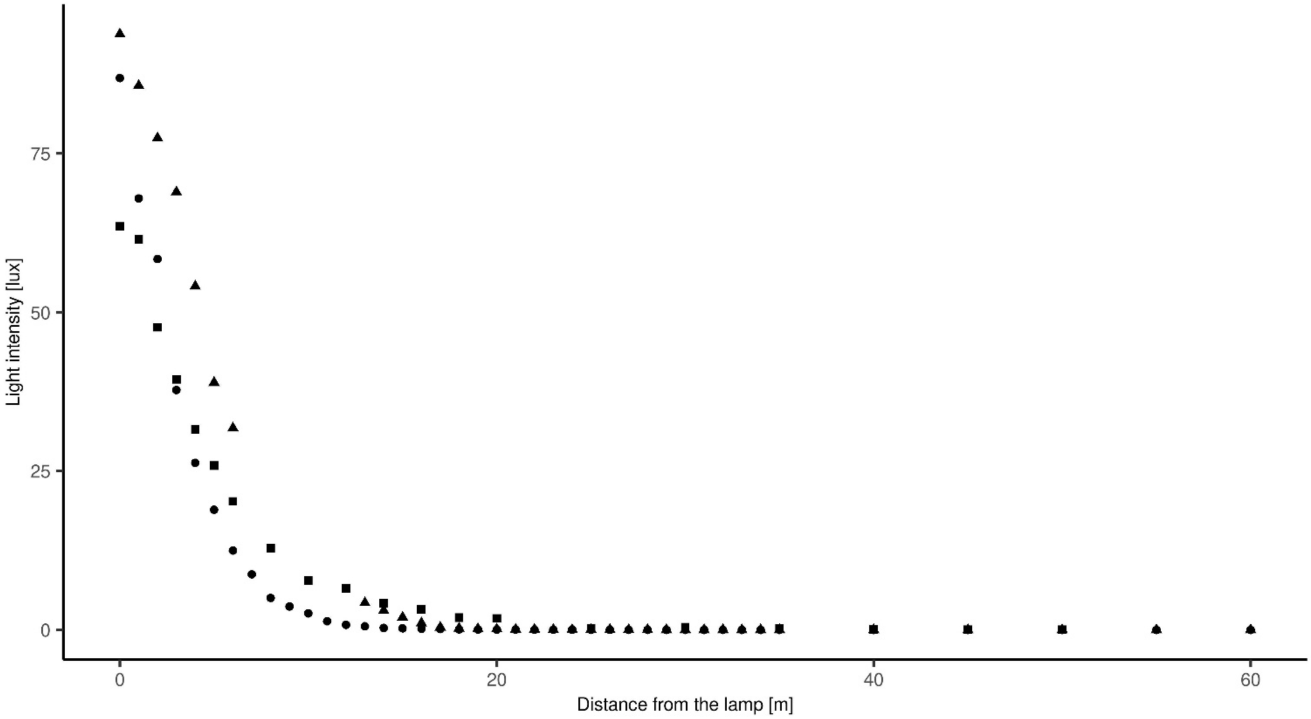

Supplement: Supplementary file 1 — Supplementary Information [file 41467_2021_22011_MOESM1_ESM.pdf]
