# Supplementary material for: Impact of artificial light at night on diurnal plant-pollinator interactions
Source: Nat Commun. 2021 Mar 16;12:1690. doi: 10.1038/s41467-021-22011-8 (PMC7966740; doi:10.1038/s41467-021-22011-8)
Supplement: Supplementary file 3 — Description of Additional Supplementary Files [file 41467_2021_22011_MOESM3_ESM.pdf]

Description of Additional Supplementary Files

File Name: Supplementary Data 1
Description: Results of the effect of artificial light at night on plant-pollinator interactions. Summary output of the general linear mixed effects model testing for the effect of artificial light at night (control versus illuminated), plant abundance (scaled logarithm), plant species (21 levels), insect group (3 levels: Diptera, Hymenoptera and Coleoptera) and the interactions between treatment, plant species and insect groups on the number of plant-pollinator interactions (response variable, logtransformed).
