# Supplementary material for: Impact of artificial light at night on diurnal plant-pollinator interactions
Source: Nat Commun. 2021 Mar 16;12:1690. doi: 10.1038/s41467-021-22011-8 (PMC7966740; doi:10.1038/s41467-021-22011-8)
Supplement: Supplementary file 5 — Reporting Summary [file 41467_2021_22011_MOESM5_ESM.pdf]

Corresponding author(s): Eva Knop

Last updated by author(s): Feb 15, 2021

Reporting Summary

Nature Research wishes to improve the reproducibility of the work that we publish. This form provides structure for consistency and transparency in reporting. For further information on Nature Research policies, see our Editorial Policies and the Editorial Policy Checklist.

Statistics

For all statistical analyses, confirm that the following items are present in the figure legend, table legend, main text, or Methods section.

n/a	Confirmed	
☐	☒	The exact sample size (*n*) for each experimental group/condition, given as a discrete number and unit of measurement
☐	☒	A statement on whether measurements were taken from distinct samples or whether the same sample was measured repeatedly
☐	☒	The statistical test(s) used AND whether they are one- or two-sided<br>*Only common tests should be described solely by name; describe more complex techniques in the Methods section.*
☐	☒	A description of all covariates tested
☐	☒	A description of any assumptions or corrections, such as tests of normality and adjustment for multiple comparisons
☐	☒	A full description of the statistical parameters including central tendency (e.g. means) or other basic estimates (e.g. regression coefficient) AND variation (e.g. standard deviation) or associated estimates of uncertainty (e.g. confidence intervals)
☐	☒	For null hypothesis testing, the test statistic (e.g. *F*, *t*, *r*) with confidence intervals, effect sizes, degrees of freedom and *P* value noted<br>*Give P values as exact values whenever suitable.*
☒	☐	For Bayesian analysis, information on the choice of priors and Markov chain Monte Carlo settings
☒	☐	For hierarchical and complex designs, identification of the appropriate level for tests and full reporting of outcomes
☐	☒	Estimates of effect sizes (e.g. Cohen's *d*, Pearson's *r*), indicating how they were calculated

Our web collection on statistics for biologists contains articles on many of the points above.

Software and code

Policy information about availability of computer code

Data collection	No software was used in the data collection.
Data analysis	R version 4.0.3 was used for data management and analysis. Libraries used: lme4, lmerTest and ggplot2.

For manuscripts utilizing custom algorithms or software that are central to the research but not yet described in published literature, software must be made available to editors and reviewers. We strongly encourage code deposition in a community repository (e.g. GitHub). See the Nature Research guidelines for submitting code & software for further information.

Data

Policy information about availability of data

All manuscripts must include a data availability statement. This statement should provide the following information, where applicable:

- Accession codes, unique identifiers, or web links for publicly available datasets
- A list of figures that have associated raw data
- A description of any restrictions on data availability

https://zenodo.org/record/4540407#.YCqYPTKg82w

Field-specific reporting

Ecological, evolutionary & environmental sciences study design

All studies must disclose on these points even when the disclosure is negative.

Study description	To experimentally test whether artificial light at night can alter plant-pollinator interactions during daytime we planned a factorial sampling design in which we surveyed 12 independent meadows. By experimentally illuminating six of them with commercial LED street lamps and leaving undisturbed the other six, we set up a two factors light treatment (dark and illuminated). The other factors were plant species (21 levels) and insect groups (three levels: Diptera, Coleoptera and Hymenoptera). Additionally we included a quantitiative variable for the abundance of each plant species on each meadow for each sampling.<br>Dependent variable was the logarithm of the number of interactions between an insect belonging to one of the three above mentioned groups and a flower of a plant belonging to one of the 21 species during one sampling. An interaction was considered as such when the insect was actively touching the sexual parts of a receptive flower.<br>We then analyzed two models: both included all the four above mentioned independent variables but one included all possible interactions among plant species, treatment and insect group, while the the second only the interaction between plant species and treatment.<br><br>To garantee equal environmental conditions each dark meadow was paired with an illuminated meadow and sampled simultaneously. I total we performed 37 sampling events within one season (from June to September 2016) in which one pair of meadows was sampled. Sampling five pairs six times and one pair seven times we obtained 2384 plant-pollinator interactions.
Research sample	We focused on the most abundant pollinator groups, namely Hymenoptera (984 interactions), Diptera (1119) and Coleoptera (281). We only used adults and collected with no regard for the sex. These three pollinator groups are meant to represent the pollinator community active during day in the considered system.
Sampling strategy	We did not predetermine a minimal sample size, but we rather maximized it by equally surveying all of our meadows a maximum number of times (6-7 per year) from the beginning to the end of the flowering season, namely from June to September.<br>Logistical reasons prevented us to sample more intensively or for more years. However, we could collect a considerable amount of plant-pollinator interactions (2384).
Data collection	Sampling was performed by walking a predetermined transect (100 meters long) across a meadow and collecting all pollinators visiting a receptive flower for later identification. The plant species, on the other hand, was noted immediately. Each walk on the transect lasted 30 minutes and we repeated it 6 times continuously till the end of one sampling event, which hence lasted 3 hours in total. Always a pair of control and treatment sites was sampled simultaneously to minimize the influence of factors other than our treatment. Typically one person worked on each meadow. However, since twelve persons participated to the data collection and not all started at the same time, often two people were present on one meadow, one teaching and the other practicing. Those persons are: Simone Giavi, Leana Zoller, Elio Herzog, Andreas Brunner, Coralie Lavaud, Ivana Cervenka, Olivia, Küchler, Laurence Etter, Sara Giovanettina, Malte Scheurer, Muriel Niederost and Perrine Huber.<br>Pollinators were captured with a hand net and then stored in plastic vials in a portable cool box.
Timing and spatial scale	The 12 meadows on which we worked for this study were all spatially independent and distributed in two areas of which one had a size of 20 square kilometers and the other about 5 square kilometer (about 17 km away from the first area). All data was collected during the time when most flowers were flowering. The first sampling was performed 01. June 2016 and the last 06. September 2016. Our goal was to maximize the amount of days between one sampling and the other on the same meadow in order to give enough time to the community to recover from the disturbance associated with the sampling itself. To do so we initially randomized the order with which meadows pairs were sampled and we kept that order for the whole season. In this way, before coming back to a given meadow pair we had first to sample all the others. The result was that each meadow pair was sampled in average every 15 days. Regarding the spatial scale, each meadow pair was at least 1 km away from another one and within pair the minimum distance was 500 m. The latter condition was not respected for one pair, but a high hill separated the two meadow making them independent. Meadows differed in size, but they had at least to comfortably include a 100 m transect that was sampled one meter on each side, meaning 200 m^2. All meadows were bigger than that.
Data exclusions	No data was excluded.
Reproducibility	The field study could be repeated, but we did not do so yet.
Randomization	At the beginning of the sampling season we randomized the order on which meadows were sampled.
Blinding	Blinding was not required since we did not assigned subjects to a treatment. Rather, we imposed a treatment and observed how the subjects (insects and plants) reacted.

Did the study involve field work? ☒ Yes ☐ No

Field work, collection and transport

Field conditions	We selected hot and sunny days that favour plant-pollinator interactions. On the other hand, we avoided rainy and windy days. Mean temperature during sampling was 25°C and cloud cover 35%. We avoided rainy days.
Location	The surveyed meadows were scattered in the area included in the rectangle which corners corresponded to the following coordinates: 46°36'31.663''N 7°23'06.438''E, 46°36'14.949''N 7°37'45.101''E, 46°48'13.896''N 7°37'28.769''E, 46°48'24.100''N 7° 22'56.285''E. The average altitude of the sampled meadows was 800 m.a.s.l.

Access & import/export	All sampling sites were accessible by car using public roads. All landowners and municipalities were extensively informed about our research activities and all work was done with due authorizations. The samples were small vials that fitted in a cool box and were therefore easily transportable.<br>Contacts of the issuing authorities: Nicole Dahinden (Naturpark Gantrisch, info@gantrisch.ch), Hanspeter Bürki (Municipality of Diemtigen, info@diemtigen.ch) and Hanspeter Luginbühl (Bernese Cantonal Forest service, sfb@be.ch).
Disturbance	We minimized disturbance to the plant and pollinator community by maximizing the time between samplings and avoiding stepping outside the predefined transects.

Reporting for specific materials, systems and methods

We require information from authors about some types of materials, experimental systems and methods used in many studies. Here, indicate whether each material, system or method listed is relevant to your study. If you are not sure if a list item applies to your research, read the appropriate section before selecting a response.

Materials & experimental systems

n/a	Involved in the study	
☒	☐	Antibodies
☒	☐	Eukaryotic cell lines
☒	☐	Palaeontology and archaeology
☐	☒	Animals and other organisms
☒	☐	Human research participants
☒	☐	Clinical data
☒	☐	Dual use research of concern

Methods

n/a	Involved in the study	
☒	☐	ChIP-seq
☒	☐	Flow cytometry
☒	☐	MRI-based neuroimaging

Animals and other organisms

Policy information about studies involving animals; ARRIVE guidelines recommended for reporting animal research

Laboratory animals	This study did not involve laboratory animals.
Wild animals	For this study we collected insects belonging to the orders of Hymenoptera, Diptera and Coleoptera. In order to identify them at least until family level, they were captured with hand net, moved to a plastic or glass vial and stored in a portable cool box containing ice until the end of the sampling event. After the sampling, the insects were brought to the institute and killed by putting them in a freezer at -20°C.
Field-collected samples	The study did not involve samples collected from the field, except for what we describe in the section on wild animals.
Ethics oversight	No ethical approval was required to collect insects from the field.

Note that full information on the approval of the study protocol must also be provided in the manuscript.
